# Pediatric ureteral stenting: state-of-the-art review

**DOI:** 10.1007/s00345-026-06347-8

**Published:** 2026-04-09

**Authors:** Abdullah Altunhan, Selim Soyturk, Thomas R. W. Herrmann, Vineet Gauhar, Theodoros Tokas, Sajid Sultan, Anna Bujons, M. Selcuk Silay, Bhaskar Kumar Somani, Selcuk Guven

**Affiliations:** 1 School of Medicine, Department of Urology, Necmettin Erbakan University, Konya, Turkey; 2 European Association of Urology Section of Endourology, Arnhem, The Netherlands; 3 Department of Urology, Kantonspital Frauenfeld, Spital Thurgau AG, Frauenfeld, Switzerland; 4 Department of Urology, Ng Teng Fong General Hospital, Singapore , Singapore; 5 Asian Institute of Nephrology and Urology, Chennai, India; 6 University General Hospital of Heraklion, Heraklion, Greece; 7 Department of Urology, A part of European Urology, Training and Research in Urological Surgery and Technology (T.R.U.S.T.)-Group, Amsterdam, The Netherlands; 8 Department of Pediatric Urology, Sindh Institute of Urology and Transplantation (SIUT), Karachi, Pakistan; 9 Division of Pediatric Urology, Urology Department Fundació Puigvert Universitat Autonòma Barcelona, Barcelona, Spain; 10 Medipol Acıbadem Hospital, Clinic of Pediatric Urology, Istanbul, Turkey; 11 Department of Urology, University Hospital Southampton, NHS Trust, Tremona Road, Southampton, SO16 6YD UK

**Keywords:** Ureteral stent, Double J stent, Children, Pediatric, Infant, Ureteroneocystostomy, Ureterorenoscopy, ESWL, Vesicoureteral reflux

## Abstract

**Purpose:**

In this scoping review, we mapped the available clinical evidence on the use of double-J (DJ) ureteral stents in pediatric patients across reconstructive, stone-related, and other endourological indications, synthesizing contemporary data on techniques, sizing, dwell time, removal strategies, outcomes, and emerging technologies.

**Methods:**

A scoping review was conducted in accordance with the PRISMA Extension for Scoping Reviews (PRISMA-ScR). The review protocol was prospectively registered in PROSPERO (CRD420251147003). PubMed, Scopus, Cochrane Library, and Web of Science were searched without date limits to 9 September 2025. Original clinical studies on double-J (DJ) ureteral stents in children were screened, data were extracted into a harmonized database, and risk of bias was appraised using RoB 2, ROBINS-I, or the Joanna Briggs Institute checklist as appropriate.

**Results:**

Fifty studies (2003–2025) were included. In reconstruction, internal DJ and externalized stents achieved similar success after pyeloplasty; externalized options commonly enabled office removal without general anesthesia (GA) but often increased length of stay and/or operating time. During ureteroneocystostomy for vesicoureteral reflux, routine stenting was associated with worse adjusted short-term outcomes; these findings are consistent with selective use. In stone disease, routine pre-stenting before ureteroscopy or extracorporeal shock-wave lithotripsy did not improve stone-free rates and increased infectious morbidity; when performed, a short dwell time (~ 2 weeks) was adequate. Across indications, modifiable drivers of morbidity included prolonged dwell, bilateral placement, and multiple lifetime stents. Practical aids included the “Age + 10 cm” length rule and strategies that reduce GA exposure (e.g., stent-on-string with disciplined protocols). Magnetic DJ systems showed high outpatient retrieval success with familiar complication profiles, while anti-biofilm/anti-encrustation coatings remain promising but require pediatric clinical validation.

**Conclusion:**

Pediatric ureteral stenting practices vary widely across indications. The mapped literature suggests broadly comparable success between internal and externalized stents in reconstruction, while highlighting the importance of dwell time, anesthesia exposure, and individualized decision-making. In stone disease, routine pre-stenting does not appear to confer consistent benefit. Overall, careful patient selection, planned dwell duration, and structured follow-up remain central to optimizing outcomes, while prospective multicenter studies are needed to strengthen the evidence base.

**Supplementary Information:**

The online version contains supplementary material available at 10.1007/s00345-026-06347-8.

## Introduction

 In pediatric urology, ureteral stents are primarily used to provide urinary drainage and anastomotic support during reconstruction for UPJ obstruction, to support pre- and post-procedural management of stone disease around URS and ESWL, to achieve urgent decompression of obstructive uropathy, and in selected congenital anomalies. In children, small ureteral calibre, growth, infection risk, and the need to minimize anesthetic exposure and family burden make direct extrapolation from adult practice unreliable; contemporary overviews therefore recommend tailoring to indication, age, anatomy, and center expertise [1, 2].

Stents are routinely used in reconstructive surgery—especially pyeloplasty for UPJ obstruction and ureteroneocystostomy (UNC)—to maintain patency across the repair and limit early edema or leakage. Options include internal double-J (DJ) stents and various externalized splints, each with trade-offs in removal setting, device care, and symptoms. New approaches (e.g., magnetic-end retrieval, stent-on-string) aim to enable office removal without general anesthesia (GA), but adoption depends on patient size, surgical approach, and institutional protocols [1, 3].

Beyond reconstruction, stents are used for stone procedures—with differing roles in URS and ESWL—and other endourological settings: temporizing drainage in selected infants with primary hydronephrosis, short-term splintage after endoscopic ureterocele incision/deroofing, adjuncts in vesicoureteral reflux (VUR) surgery, and urgent decompression of obstructive uropathy as an alternative to percutaneous nephrostomy (PCN). Practice varies in timing, insertion route (retrograde/antegrade/transrenal), stent caliber/length, dwell time, and removal strategy [2, 4, 5].

Given the variability, this review categorizes pediatric ureteral stenting by indication into three groups: reconstruction (pyeloplasty, UNC), stone disease, and other endourological conditions (ureterocele, VUR surgery, congenital hydronephrosis). We focus on DJ stent practice—technique and insertion route, stent caliber/length, dwell time, and removal—and summarize outcomes relevant to children and families (clinical success, complications, reintervention, anesthesia burden, cost) consistent with European guidance [2]. Accordingly, this review focuses on clinical studies evaluating double-J (DJ) ureteral stents in children, with externalized drainage devices considered only when directly compared with DJ stents.

## ​Methodology

This scoping review was conducted in accordance with the Preferred Reporting Items for Systematic Reviews and Meta-Analyses Extension for Scoping Reviews (PRISMA-ScR) [6]. The review protocol was prospectively registered in the International Prospective Register of Systematic Reviews (PROSPERO; CRD420251147003). The objective of this scoping review was to map the available clinical evidence on pediatric double-J ureteral stenting, describe patterns of use and reported outcomes, and identify knowledge gaps, without performing quantitative synthesis.

### Search strategy

Literature searches were conducted in PubMed, Scopus, Cochrane Library, and Web of Science without date limits, up to September 9, 2025. The aim was to identify all studies reporting the use of DJ ureteral stents in pediatric patients, irrespective of indication.

### Core query across platforms

(“ureteral stent” OR “ureteric stent” OR “double J stent” OR “DJ stent”) AND (“child” OR “children” OR “pediatric” OR “paediatric” OR “adolescent” OR “infant”).

In PubMed, MeSH terms were combined with free text; analogous controlled vocabularies were used in other databases.

Five authors independently reviewed the strategy. The search retrieved 868 PubMed, 1,252 Scopus, 111 Cochrane Library, and 780 Web of Science records; five additional articles were found by manual search.

### Screening and selection

The initial search identified 3,011 records. After removing 1,356 duplicates, 1,660 records remained for screening. Titles and abstracts were screened for relevance to pediatric double-J stent use by two reviewers; a third reviewer adjudicated discrepancies. Screens were cross-checked for accuracy, and disagreements were resolved in consensus meetings.

**Fig. 1:**
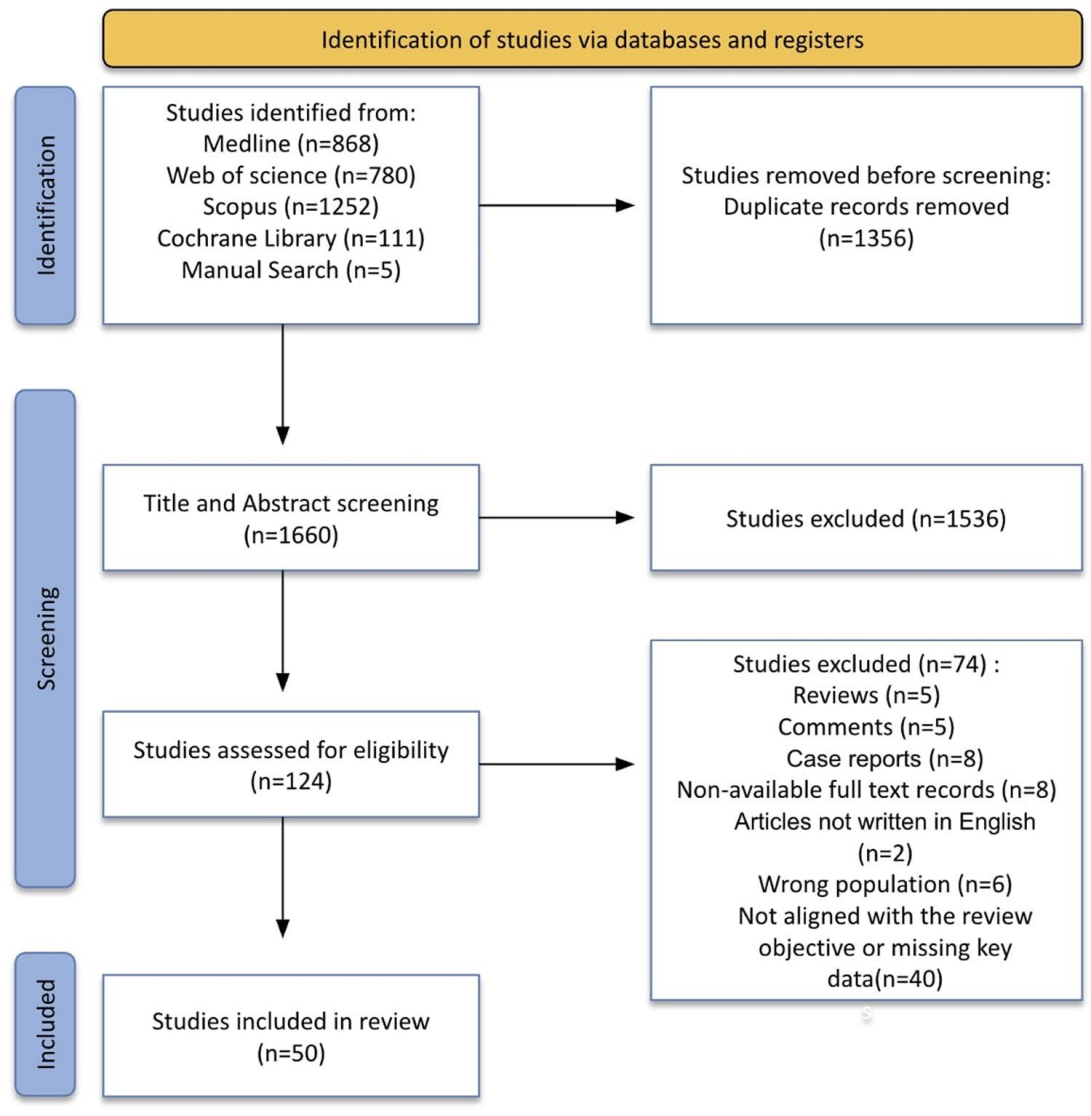
PRISMA-ScR flow diagram of the scoping review

Studies were eligible if they reported clinical outcomes of internal double-J (DJ) ureteral stents in pediatric patients. Studies focusing exclusively on external drainage methods or non-DJ ureteral stents were excluded, unless these devices were directly compared with DJ stents. Animal studies, in vitro experiments, case reports, technical notes without clinical outcome data, and narrative reviews were excluded. When reported, data were interpreted with attention to age subgroups (infants, young children, and adolescents) to avoid overgeneralization across heterogeneous pediatric populations. In total, 1,536 records were excluded at the title/abstract stage as irrelevant. The remaining 124 records underwent full-text assessment focused on original clinical studies of pediatric DJ stent use. Seventy-four were excluded reviews (*n* = 5), comments (*n* = 5), case reports (*n* = 8), no full text (*n* = 8), non-English (*n* = 2), wrong population (*n* = 6), or not aligned with the review objective/missing key data (*n* = 40). Fifty studies met the inclusion criteria and were included in the qualitative synthesis. The process is shown in the PRISMA-ScR flow diagram (Fig. 1).

### Data extraction and synthesis

A database was created to capture study characteristics (design, country, population), indication, surgical approach, stent type/size, insertion route, dwell time, and outcomes. Entries were double-checked and harmonized by the researchers. Studies were categorized into three groups: reconstruction, stone disease, and other endourological procedures. Results were synthesized narratively to provide an overview of current practice.

### Study qualification

**Fig. 2:**
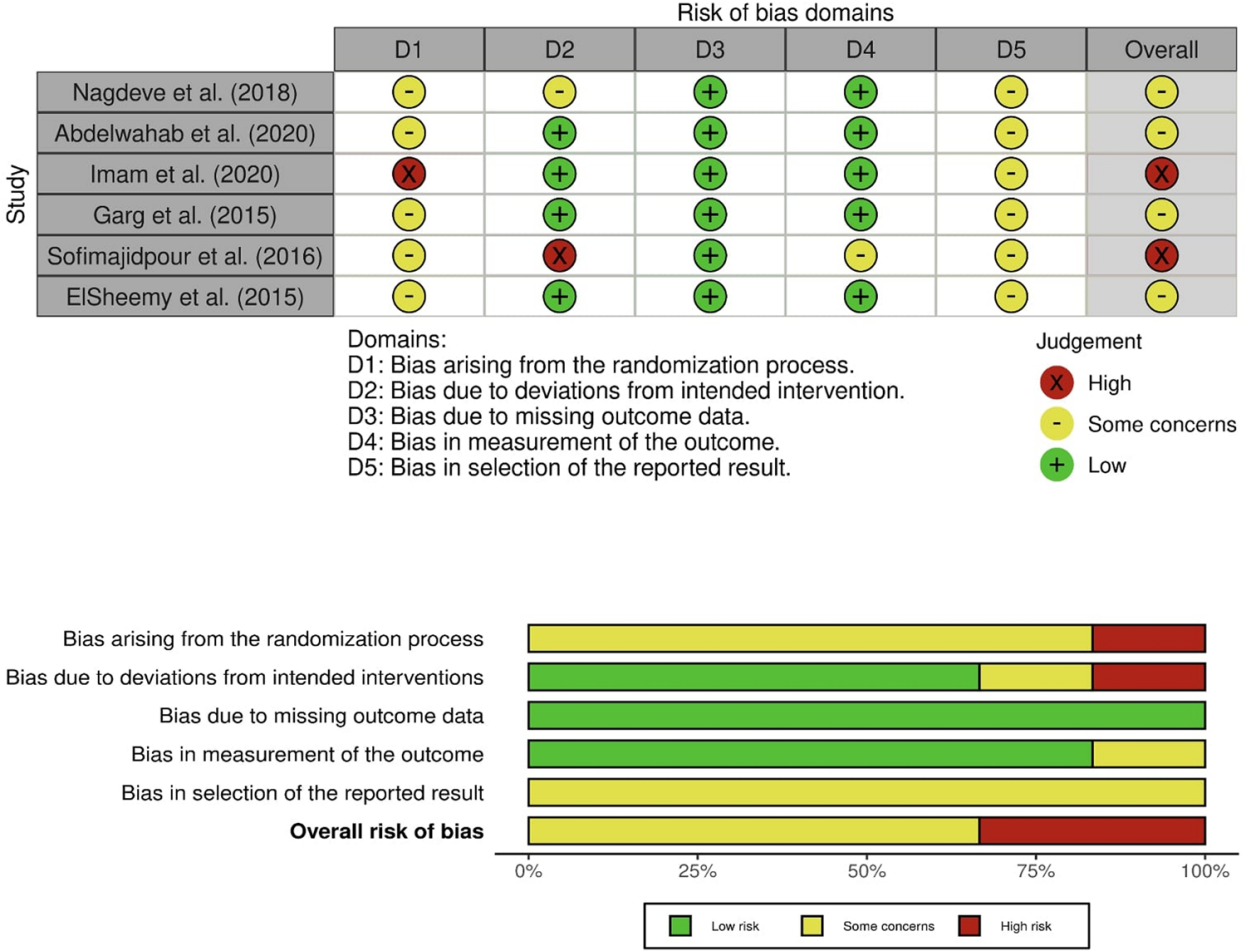
ROB-2 quality assessment of included studies

**Fig. 3:**
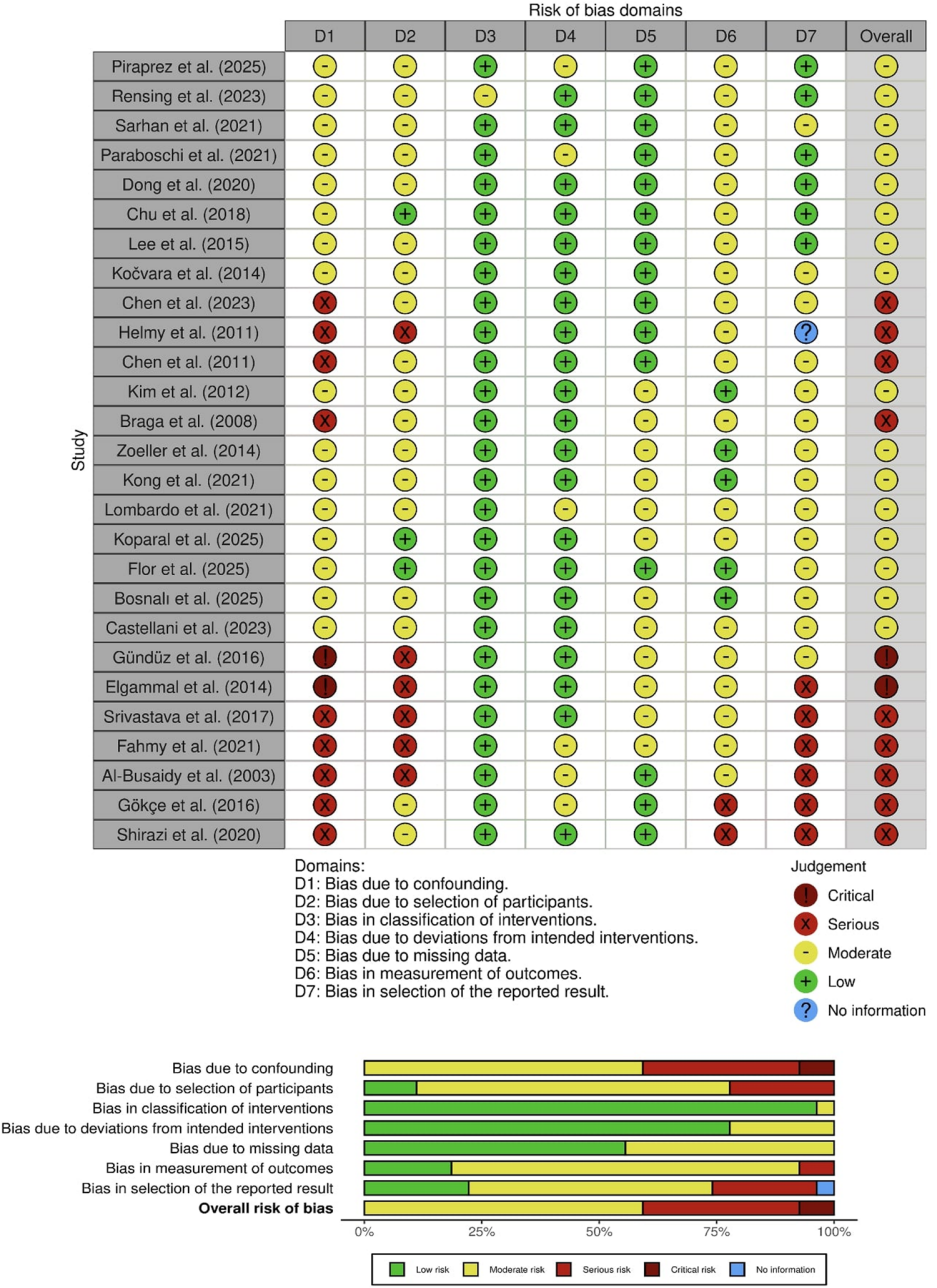
ROBINS-I quality assessment of included studies

**Fig. 4:**
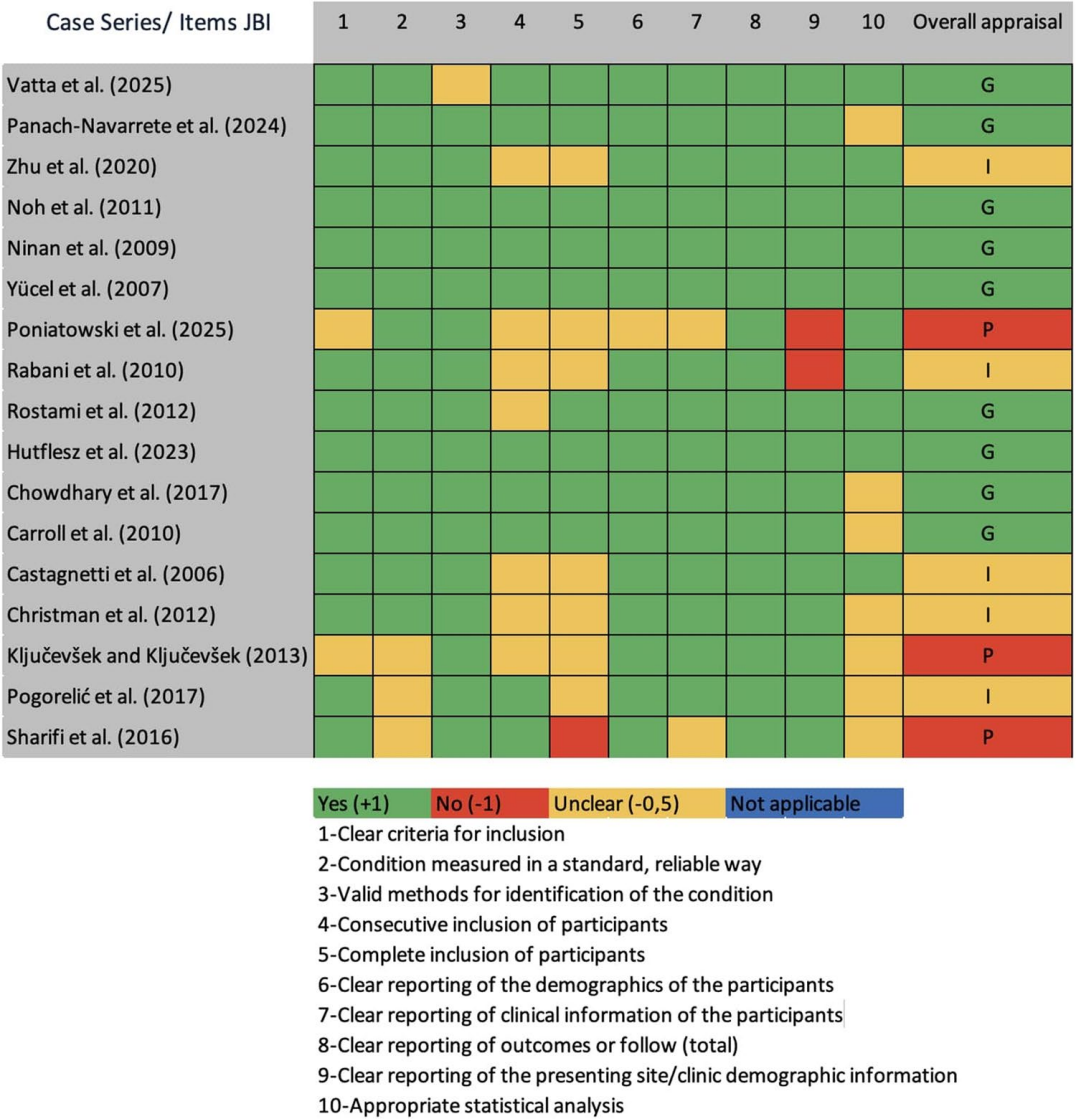
JBI quality assessment of included studies

The following tools were used: Risk of Bias 2 (RoB 2) for randomized trials; Risk Of Bias In Non-randomized Studies of Interventions (ROBINS-I) for nonrandomized studies; and Joanna Briggs Institute (JBI) case-series checklist for single-arm reports [7, 8]. Studies were appraised by domain and mapped to a three-level risk-of-bias scale (low, moderate, high) for cross-design synthesis. Visualizations followed tool conventions (see Figs. 2, 3, 4).

## Results

Findings are presented as a narrative synthesis describing ranges, patterns, and trends across heterogeneous studies rather than comparative effect estimates.

Fifty studies (2003–2025) from North America, Europe, and Asia were included and grouped as reconstruction, stone disease, and other endourological procedures [9–58]. Designs spanned randomized/prospective trials, nonrandomized cohorts, single-arm series, and database/technical reports; most were retrospective, single-center. Stent strategies were diverse: internal DJ and externalized options—pyeloureteral (PU), externalized pyeloureteral (EPU), cutaneous pyeloureteral (CPU), single-incision pyeloplasty stent (SIPS), and trans anastomotic external ureteral splint (TEUS). Insertion route, caliber/length, dwell, and removal logistics (office vs. operating room (OR); with/without GA) varied widely.

### Reconstructive procedures

#### Pyeloplasty for UPJ obstruction

Across pyeloplasty studies, “success” was variably defined but most commonly included a combination of symptomatic improvement, radiological resolution or improvement of hydronephrosis (on ultrasound or diuretic renography), absence of reintervention, and preservation of renal function. Overall, across reconstructive studies, internal and external stenting strategies were associated with similarly high reported success rates, whereas differences were observed primarily in perioperative burden, anesthesia exposure, dwell time, and resource utilization. The body of evidence in this subsection consisted predominantly of retrospective single-center cohorts, with a smaller number of prospective and randomized studies, and overall demonstrated a moderate-to-high risk of bias.

Across open, laparoscopic, and robotic series comparing DJ with externalized stents, similar success was observed. In a multicenter open cohort, success—defined as symptom relief plus improvement on hydronephrosis or diuretic renogram and no reintervention—was reported in approximately 95–97% of cases for both DJ and PU stents [13]. In a laparoscopic cohort, success defined as clinical improvement confirmed by imaging without redo surgery was comparable between DJ and EPU; however, dwell time was substantially longer with DJ stents (mean 62 ± 30 days) compared with EPU (8 ± 3 days), and removal costs were lower for EPU (£687 vs. £1,426) [14]. In another study, success defined as ultrasound anteroposterior pelvic diameter reduction without reoperation was similar between DJ and TEUS, with longer dwell times for DJ (~ 30 days) compared with TEUS (5–7 days). TEUS was initially associated with longer operative time and length of stay (LOS), although this difference diminished after standardizing removal at day 5; no urine leaks were reported in either group [15].

During robotic-assisted laparoscopic pyeloplasty (RALP), success—defined as stabilization or improvement of hydronephrosis without redo surgery—was comparable between EPU and DJ stents, with reported success rates of approximately 94–95% [17]. EPU stents were removed in the clinic without general anesthesia (GA), while length of stay (LOS) was modestly longer than with DJ stents. In a cohort comparing unstented repairs, DJ stents, and transanastomotic external stents, success—defined as relief of obstruction without late failure—was similar across groups. LOS tended to be longest with externalized stents (approximately 8 days) compared with DJ-stented and unstented repairs (approximately 3–4 days), and DJ stenting was associated with a higher number of anesthetic episodes due to the need for stent removal [20]. In studies comparing EPU with DJ stents, success—defined as improvement in hydronephrosis or symptoms without redo—was comparable; DJ stents generally required GA for removal, whereas EPU stents were removed on an outpatient basis [19]. Transrenal external stents were associated with fewer stent-related complications and fewer redo pyeloplasties than DJ stents but were accompanied by longer operative times and longer LOS [32]. Comparisons between DJ stents and single-incision pyeloplasty stents (SIPS) demonstrated near-identical success rates (approximately 95%), with SIPS enabling office-based removal, lower overall costs, and similar complication rates [31].

During RALP, success—defined as stable or improved hydronephrosis without redo surgery—was comparable between DJ and CPU stents, with reported success rates of approximately 96–100% [36]. DJ stents were associated with more frequent intravenous opioid use and an almost universal need for GA for stent removal, whereas CPU stents were typically removed without GA. Postoperative UTI were reported more often in DJ-stented patients [36]. After laparoscopic pyeloplasty, the addition of a short external pyelostomy to a standard DJ stent resulted in comparable overall success, defined as absence of reoperation or worsening hydronephrosis. This approach was associated with fewer non-catheter–related complications, shorter LOS, and shorter drain duration compared with DJ stenting alone [21].

Randomized trials demonstrated similar overall patterns. In open pyeloplasty, stentless and DJ approaches showed comparable early radiographic improvement (approximately 80%), while DJ use was associated with longer operative times, more irritative symptoms, and a greater number of analgesic days; length of hospital stay was similar between groups [16]. After laparoscopic pyeloplasty, a 1-week tethered DJ achieved patency at follow-up comparable to a 4-week DJ, with all cases reported as unobstructed at 6 months. Short-term stenting was associated with fewer postoperative UTI and irritative symptoms and enabled office-based removal without GA [22].

Placement and verification were consistently reliable across techniques, with rapid antegrade positioning and high confirmation rates. Externalized stents avoided the need for an additional anesthetic for removal, whereas DJ stents typically required GA. Antegrade placement success was reported at approximately 90%, with positioning achieved in under 5 min and no recurrent obstruction [23]. Intraoperative confirmation rates reached 100% using different verification methods, with ultrasound providing faster confirmation and requiring less bladder filling than alternative approaches; overall pyeloplasty success was maintained on follow-up [11]. No placement failures were reported for either externalized transanastomotic or internal DJ stents, with GA required only for DJ removal [24]. Modified antegrade techniques using cystoscopic guidance improved correct positioning compared with conventional methods, with all patients demonstrating improvement at 12 months [29].

Routine ~ 4-week DJ use after laparoscopic pyeloplasty in a large single-center series (*n* = 1,349) was associated with low device-related events (3.04%; blockage 1.85%), while UTI (17.6%) and flank pain (12.5%) were frequent during dwell; removal succeeded in 96.96% at 24–33 days [18]. A process-improvement initiative by Poniatowski et al. reduced RALP stent-insertion time from ~ 14 to 12 min (to 8 min excluding one anatomic outlier) through workflow standardization and dedicated tools [27].

Comparative cohorts informed drainage selection. In a large laparoscopic pyeloplasty series, recurrence or reoperation rates were reported more frequently with TEUS than with DJ or percutaneous PU, alongside higher overall complication rates and longer LOS and operative time for externalized drainage options [34]. In open pyeloplasty, short-term and long-term DJ stenting were both associated with high 6-month recovery rates, while shorter dwell time was accompanied by fewer reported UTI, lower rates of encrustation, and less stent migration [28].

In a single-center RALP protocol using a DJ stent with a retrieval string to enable office-based removal, lower 30-day costs were reported compared with a national comparator. However, a higher rate of clinical pyelonephritis was also observed in this cohort [12].

Two contemporary cohorts evaluated magnetic-tip DJ stents in pediatric pyeloplasty. Antegrade placement was frequently unsuccessful, with reported antegrade success of approximately 48% in the MEDJUS cohort, varying by stent size. When successfully placed, office-based removal was achieved in most cases. In the cohort reported by Vatta et al., stent insertion was successful in approximately three-quarters of patients, while combined success defined as successful insertion followed by office removal without GA was reported in just over half of cases. Retrieval failures were more commonly reported after retroperitoneal approaches and in younger or smaller children [9, 10].

In open pyeloplasty, routine internal DJ stenting was reported to be associated with very low early morbidity, with no urine leak, UTI, or re-obstruction observed, and a median LOS of approximately 2 days [25]. After laparoscopic pyeloplasty, use of a short dwell “dangler” DJ was associated with high reported success; however, reintervention was required in a subset of patients and irritative symptoms were commonly noted [26].

In an age-matched cohort, outcomes after stented and unstented repair were reported to be similar. APPD reduction and rates of re-obstruction were comparable between groups, and no urine leaks or febrile UTI were observed in either cohort [30]. In a randomized open pyeloplasty trial, DJ stenting and nephrostomy were both associated with comparable short-term functional improvement. DJ use was accompanied by a shorter hospital stay, whereas nephrostomy was associated with fewer minor device-related symptoms and lower reported overall costs [33]. In infants, use of a closed-end 3 F DJ allowed office-based removal at approximately 4 weeks, with no reported leaks, UTI, or displacement and a mean LOS of around 3 days [35].

When used as a standalone approach for UPJO, ureteral stenting was associated with lower reported success rates compared with pyeloplasty. In this cohort, renal function was generally preserved despite treatment failure, while in infants nearly half of cases managed with stenting alone ultimately failed [37].

#### Ureteroneocystostomy (UNC) for VUR

In a national cohort including 4,550 pediatric UNC procedures, routine DJ stenting was associated with higher rates of emergency department visits, readmissions, and postoperative UTI, as well as longer LOS and increased operative time, compared with unstented procedures [38] (see Table 1).

**Table 1:** Detailed explanation of the presented studies of reconstructive procedures

First author, year	Country	Study design (RCT, cohort, case series)	Population (n), (age range)	Surgical approach	Comparison group	Stent duration (days/weeks)	Main findings
Piraprez et al. (2025) [9]	France	RC	n=91; median 9.2 y (1.5–17.7)	RALP	MEDJUS vs DJUS	MEDJUS 4 wk (office); DJUS NA (OR/GA)	Overall pyeloplasty success 90%; MEDJUS office removal success 93.3% (14/15) but antegrade insertion failure high (overall MEDJUS success 51.8%; XS 69.2% vs S 37.5%); UTI 5.5%; GA avoided for MEDJUS removal.
Vatta et al. (2025) [10]	France	RC	n=33; median 10 y (0.6–18)	RALP	internal approach T-RALP vs R-RALP	NA (planned clinic removal, no GA)	Combined “primary success” (insertion + clinic removal without GA) 58–60%; insertion 74% (23/31); 5/23 retrieval failures—all after retroperitoneal RALP (p=0.04); no Clavien > II; median LOS 2 d.
Panach-Navarrete et al. (2024) [11]	Spain	RC	n=20; median 48 mo (5–132)	LP-TP	Ultrasound vs reflux visualization	4 wk (OR/cysto)	Both ultrasound and reflux tests verified stent 100%; pyeloplasty success 100%; bladder over-distention 50% with reflux vs 0% with ultrasound (p=0.02); one leak 5% → temporary nephrostomy.
Rensing et al. (2023) [12]	USA	RC	n=85; NA	RALP	None	NA	Adding pre-RAP retrograde DJ + external string took ~15 min; 30-day cost $10,548 vs $14,119 national cohort (−25%, p<0.001); unplanned return 15.3%, unplanned procedures 8.2%, pyelonephritis 7.1%.
Sarhan et al. (2021) [13]	Egypt and Saudi Arabia	RCC	n=175; median 24 mo (IQR 9–60; 3–144)	OP	DJ vs PU	DJ 4–12 wk (med 42 d); PU 1–2 wk (med 9 d)	Success DJ 95.5% vs PU 97% (p=0.63); LOS 3.7 vs 4.2 d (p=0.003); overall complications 8% vs 9% (NS); procedures/anesthetics markedly higher with DJ (221 vs 67, p<0.001).
Paraboschi et al. (2021) [14]	UK	RCC	n=53 op / 51 pts; mean age DJ 107.2 mo vs EPU 58.8 mo	LP-TP	DJ vs EPU	DJ 61.6 ± 30.2 d; EPU 8.1 ± 3.1 d	Success equivalent; complications DJ 15.4% vs EPU 11.1% (NS); cost of stent removal £1426 (DJ) vs £687 (EPU) (p<0.01).
Dong et al. (2020) [15]	China	RCC	n=85; median ≈46 mo	LP-TP	DJ vs TEUS	DJ ~30 d; TEUS 5–7 d (later std 5 d)	No redo in either arm; similar improvement in APRPD; TEUS had longer OR time and longer LOS initially, but LOS normalized when removal standardized to 5 d; DJ had more UTIs (text cites 5 cases, 2 re-stents).
Nagdeve et al. (2018) [16]	India	RCT	n=39; ≤12 y (min 2 mo)	OP	DJ stent vs stentless pyeloplasty	POD15 (short GA)	Resolution similar (Non-stented 80% vs Stented 78.9%); stented had +18.5 min OR time, more dysuria/spasm symptoms, longer analgesic use (7.7 vs 3.45 d); one major stent complication.
Chu et al. (2018) [17]	USA	RC	n=61; median 7.7 y (DJ) vs 1.2 y (EPU)	RALP (TP)	DJ vs EPU	DJ 45 d (IQR 41–58); EPU 34 d (19–55)	Operative success EPU 95% vs DJ 94%; EPU LOS +0.6 d (p=0.04); gross hematuria 25% (DJ) vs 0% (EPU) (p=0.03); all EPU removed in clinic (no GA) vs all DJ under GA.
Zhu et al. (2020) [18]	China	RC	n=1,349; mean 4.23 y (0–17)	LP-TP	None	~4 wk (med 29 d, IQR 26–41)	DJ removed median 29 d; device-related events 3.04% (blockage 1.85%, migration 0.67%); UTI 17.6% (median 22 d); lumbar pain 12.45%; removal successful 96.96% at 24–33 d.
Lee et al. (2015) [19]	Canada	RCC	n=62; EUP mean 40 mo vs DJ mean 80 mo	OP+LP	EUP vs DJ	DJ NA (OR/GA); EUP clinic removal	Success similar; overall complications EUP 20.8% vs DJ 5.2% (NS); LOS trend shorter with EUP (1.3 vs 1.9 d, p=0.07); all DJ needed GA for removal, EUP removed outpatient.
Kočvara et al. (2014) [20]	Czechia	RCC	Lap n=70; mean 38 mo (1–5 y) | Open hist n=52	LP-TP	Laparoscopy: L1 (unstented) vs L2 (DJ) vs L3 (external); plus historical open cohorts (unstented vs external)	Ext 8.4 d; DJ removal needs 2nd GA (dur NA)	All groups relieved obstruction; urine leaks 8.8% in unstented (all with crossing vessels); total complications L1 11.8% vs L2 14.3% vs L3 6.7%; OR time longest with external (190 min, p=0.005); LOS longest external (8.4 d, p<0.001); anesthetics: unstented 1.2, DJ 2.2, external 1.0.
Chen et al. (2023) [21]	China	RCC	n=93; mean ≈26–31 mo (similar)	LP	DJ only vs DJ + pyelostomy tube	DJ ~62 d; pyelostomy off next day if OK	Success 100% (DJ+pyelostomy) vs 92.3% (DJ); LOS 5.22 vs 6.46 d (p=0.010); non-catheter complications 2.4% vs 19.2% (p=0.03); overall complications NS.
Abdelwahab et al. (2020) [22]	Egypt	RCT	n=37; <15 y; mean 7.7 y	LP-TP	4-week DJ vs 1-week tethered (“dangler”) DJ	4 wk vs 1 wk	1-week tethered DJ non-inferior to 4-week DJ for patency; irritative symptoms 15.8% vs 61.1% (p=0.004) and UTI 10.5% vs 44.4% (p=0.029) lower with 1-week; no leaks.
Noh et al. (2011) [23]	USA	RC	n=29; mean 10 y (19 mo–18 y)	RALP	None	4–6 wk (OR/cysto)	Antegrade DJ placement success 90% (26/29); 1 leak managed conservatively; no symptomatic UTI; all stents removed at 4–6 wk under brief GA.
Helmy et al. (2011) [24]	France	RCC	n=22; mean 31 mo (EUPS) vs 37 mo (DJ)	LP-RP	EUPS vs DJ	EUPS D10–12 (clinic); DJ 4–6 wk (GA)	No failures in either group; LOS similar (~2.2 vs 2.45 d); DJ had 1 distal coil displacement; EUPS removed day 10–12 in clinic, DJ at 4–6 wk under GA.
Ninan et al. (2009) [25]	UK	RCS	n= 60 pts / ops 61; ages NA (neonate→teen)	OP	pre-simulation vs post-simulation cases	6–12 wk (day-case cysto)	In stented cases (n=58): leaks 0%, UTI 0%, displacement 0%; median LOS 2 d; overall reoperation 1.6% (occurred in unstented subset).
Yücel et al. (2007) [26]	USA / Türkiye	RCS	n=20; mean 11.3 y (4.6–17.2)	LP-TP	None	7–18 d (mean 10.3 d)	Success 89%; short dwell (~10 d) with dangler enabled awake removal; reintervention 30%; UTI 5%; bladder spasm 10%.
Poniatowski et al. (2025) [27]	USA	PSI	n=16 (pre=9, post=7); age NA	RALP	Pre-simulation vs post-simulation cases (same institution)	NA	Stent insertion time fell from 14 → 12 min overall, → 8 min excluding one outlier (trend, not statistically significant); patient safety not assessed.
İmam et al. (2020) [28]	Bangladesh	PCC	n=62; 1 mo–11.6 y (median 5 y)	OP	Short-term DJ stent vs long-term DJ stent	4 wk (28–30 d)	Success at 6 mo 97% (short-term) vs 87% (long-term) (NS); UTI 1 vs 13 (p<0.001); encrustation 0% vs 9.6%; migration 0% vs 6.4%—supports shorter dwell (~4 wk).
Chen et al. (2011) [29]	China	RCC	n=77; <5 y; mean 26.9 mo (6–60)	LP-RP	CAG vs MAG (antibiotic strategy groups)	4–6 wk (cysto)	Stent placement success 100% (MAG) vs 86% (CAG); complications 0 vs 3 (malpositions/hematuria) respectively; all showed hydronephrosis improvement at 12 mo.
Kim et al. (2012) [30]	South Korea	RCC	n=70 pts / 76 RU; mean 2.2 ± 3.8 y	OP+LP	DJ stent vs stentless	mean 31.9 ± 8.4 d	Hydronephrosis and renogram improved similarly; recurrence 3.7% (non-stented) vs 9.0% (stented) (p=0.575); no leaks or febrile UTI in either group.
Braga et al. (2008) [31]	Canada	RCC	n=470; median 18–19 mo	OP	DJUS vs SIPS (externalized DJ)	DJUS 6–8 wk; SIPS 7–10 d	Success DJUS 95.0% vs SIPS 94.7%; complications 9.9% vs 8.3% (NS); SIPS cheaper by CAD $565 and avoided second GA.
Zoeller et al. (2014) [32]	Germany	RCC	n=86; mean 5.6 y (78 d–17.3 y)	LP-TP	DJ vs transrenal externalized stent	DJ 20–73 d (mean 35.8 d, GA); Transrenal ~7 d (no GA)	Stent-related problems DJ 35% vs transrenal 13% (p<0.05); redo pyeloplasty 4 (DJ) vs 0 (transrenal); LOS 4.0 vs 7.3 d (external longer due to inpatient until removal).
Garg et al. (2015) [33]	India	RCT	n=40; 2 mo–12 y (mean DJ 2.7 y; neph 3.76 y)	OP	DJ vs nephrostomy	DJ ~2 wk (GA); nephrostomy off before discharge	Imaging outcomes similar; LOS 5.15 vs 11.95 d (DJ shorter, p=0.001); any minor complication 85% (DJ) vs 25% (nephrostomy) (p=0.0003); DJ added second GA.
Kong et al. (2021) [34]	China	RC	n=838; median (mo): DJ 57, TEUS 30, PU 48	LP	DJ vs PU vs TEUS	DJ 28.5 ± 12.2 d; PU 10.9 ± 8.2 d; TEUS 7.4 ± 1.8 d	Recurrence: TEUS 3.19% vs DJ 0.36% vs PU 0.93% (p<0.01); overall complications 12.23% (TEUS) vs 4.42% (DJ) vs 8.41% (PU) (p=0.001); OR time shortest with DJ (median 100 min).
Rabani (2010) [35]	Iran	PCS	n=12 infants; 3–10 mo (mean ~6.5)	OP	None	4 wk (skin-tether pull; no GA)	Modified “closed-end” DJ removed by skin pull at 4 wk with 0 UTIs/leaks/displacements; mean LOS 3 d in infants.
Lombardo et al. (2021) [36]	USA	RC	n=103; mean DJ 7.6 y; CPU 3.9 y	RALP	DJ vs CPU stents(cutaneous pyeloureteral external stent)	DJ 45.2 ± 25.0 d; CPU 8.3 ± 4.2 d	Success DJ 95.7% vs CPU 100% (NS); GA for removal 99% (DJ) vs 3% (CPU) (p<0.001); IV opioids 27% vs 9% (p=0.04); migration 3% (DJ) vs 15% (CPU) (p=0.03).
Koparal et al. (2025) [37]	Türkiye	RC	n=126; median 67 mo (1–201)	OP /RALP-P ; Endo-DJ	Yes — pyeloplasty vs dJ-stent	med ~10 wk	Definitive success 43.9% (DJ-stent) vs 88.4% (pyeloplasty) (p<0.001); procedure type predicted success (OR 6.87, p<0.001); complications similar; stent preserved SRF short-term (no SRF change).
Flor et al. (2025) [38]*	Canada	RC	n=4,550; 0–17 y; median 47.36 mo (68.7% female)	Open, lap/robot, or combined; intravesical/extravesical Unc	DJ stent vs stentless	≤30 d in 84%; >30 d in 16%	Stented patients had higher ED visits (OR 1.47, p=0.0019), readmissions (OR 2.52, p<0.0001), and UTI (OR 2.73, p<0.0001), longer LOS (RR 1.37, p<0.0001) and +31 min OR time.

*AP(R)PD/APPD* anteroposterior (renal) pelvic diameter, *CPU* cutaneous pyeloureteral (external stent), *d* days, *DJ* double-J ureteral stent, *DJUS* double-J ureteral stent (internal), *EPU/EUPS* externalized pyeloureteral stent (trans-anastomotic), *GA* general anesthesia, *IQR* interquartile range, *I.V.* intravenous, *Lap* laparoscopic, *Lap-RP* laparoscopic retroperitoneal, *Lap-TP* laparoscopic transperitoneal, *LOS* length of stay, *LP* laparoscopic pyeloplasty, *LP-RP* laparoscopic retroperitoneal pyeloplasty, *LP-TP* laparoscopic transperitoneal pyeloplasty, *MEDJUS* magnetic-end double-J ureteral stent, *min* minutes, *mo* months, *NA* not reported, *NS* not significant (statistical), *OP* open pyeloplasty, *OR (room)* operating room, *OR (stats)* odds ratio, *PCC* prospective comparative cohort, *PCS* prospective case series, *PSI* prospective simulation-based quality improvement (pre–post observational), *PU* pyeloureteral externalized stent (trans-anastomotic drainage), *RALP* robot-assisted laparoscopic pyeloplasty, *RC* retrospective cohort, *RCC* retrospective comparative cohort, *RCS* retrospective case series, *RCT* randomized controlled trial, *SFR* stone-free rate, *SIPS* stented internal pyeloureteral system (externalized DJ; open pyeloplasty literature), *TEUS* trans-ureterocystic external urethral stent, *TP/RP* transperitoneal/retroperitoneal, *UTI* urinary tract infection, *wk* weeks, *y* years

*All studies were for UPJO, except for Flor et al. (2025) which was for VUR

### Stone disease

In stone-related studies, success was primarily defined by stone-free status on follow-up imaging, occasionally combined with absence of secondary procedures or complications. Across stone-related studies, reported outcomes suggest that routine pre-stenting does not consistently translate into higher stone-free rates, while complication profiles, dwell duration, and patient selection appear to influence reported results. Most studies addressing stone-related indications were retrospective observational cohorts with moderate-to-high risk of bias, reflecting heterogeneity in patient selection, procedural techniques, and outcome reporting.

#### ESWL

Across pediatric ESWL cohorts, prophylactic JJ stenting was not associated with higher reported stone-free rates and was frequently accompanied by higher rates of postoperative infection. Reported stone-free rates were similar or lower in stented compared with non-stented patients across multiple series [39, 41, 42]. Earlier series focusing on staghorn calculi reported comparable stone clearance between stented and non-stented approaches, while major obstructive or septic events and length of hospital stay were reported less frequently in stented patients [47]. In infants, ESWL achieved high final clearance rates, and pre-stenting was described as part of the management strategy in selected cases with larger stone burdens to mitigate post-procedural obstruction [48].

#### URS/RIRS

In a multicenter pediatric RIRS cohort, pre-stenting was not associated with higher reported stone-free rates, while higher rates of febrile UTI and hematuria were reported among pre-stented patients compared with those who were not pre-stented [40]. Single-center URS series reported higher single-session success rates in pre-stented patients, particularly for ureteral stones and larger stone burdens, with similar overall complication rates [49]. When pre-stenting was performed before pediatric RIRS, extending dwell time from 2 to 4 weeks was not associated with higher reported stone-free rates or shorter operative time, while higher rates of postoperative UTI were observed with longer dwell [46]. In cohorts undergoing difficult primary URS, a strategy involving short-term pre-stenting followed by secondary URS was associated with reduced need for active dilation, shorter operative time, and higher reported stone-free rates [44]. For initial decompression in obstructive anuria due to bilateral ureteric stones, both JJ stenting and PCN were associated with similar renal recovery times. JJ stenting was associated with fewer overall complications and facilitated subsequent definitive endoscopic management, whereas PCN was more commonly used in patients with larger stone burdens [43].

#### Laparoscopic ureterolithotomy

Stentless laparoscopic ureterolithotomy was reported to achieve complete stone clearance and was associated with fewer stent-related symptoms compared with DJ-stented procedures. Two brief urine leaks were described and resolved conservatively, and DJ stenting was used selectively in cases with markedly inflamed ureteral mucosa [45] (see Table 2).

**Table 2:** Detailed explanation of the presented studies of stone disease

First author, year	Country	Study Design (RCT, cohort, case series)	Population (n), (age range)	Surgical approach	Comparison group	Stent duration (days/weeks)	Main Findings
Bosnalı et al. (2025) [39]	Türkiye	RC	n=268 pts / 277 RU; ≤18 y; mean 7.3 ± 4.8 y	ESWL	Pre-stented vs non-pre-stented	Removed within 6 weeks if no obstructing fragments	Pre-ESWL DJS did not improve SFR (60.6% vs 68.4%, p=0.36) nor complications; steinstrasse 12.1% vs 12.3%; reintervention ~68% when steinstrasse occurred.
Castellani et al. (2023) [40]	Italy, UK, Türkiye, Spain, Singapore, India, Nepal, Hong Kong, France	RCC	n=389; <18 y; mean 8.30 vs 10.43 y (pre-stent vs none)	RIRS	Pre-stented vs non-pre-stented	10–14 d prestent (passive dilation)	SFR similar (residuals 26.4% prestent vs 30.7% no-prestent, p=0.322); infections higher with prestent (febrile UTI 10.7% vs 3.7%, p=0.016); hematuria 7.1% vs 1.5% (p=0.018).
Gündüz et al. (2017) [41]	Türkiye	RCC	n=20; mean 4.5 y vs 4.0 y	ESWL	DJ-stented vs unstented ESWL	3 wk	SFR 80% (stented) vs 100% (unstented) (NS); minor LUTS in both; no steinstrasse; small sample.
Sofimajidpour et al. (2016) [42]	Iran	RCT	n=68; mean 4.55 y vs 4.13 y (<13 y)	ESWL	DJ-stented vs unstented ESWL	NA	SFR 73.5% (stent) vs 85.3% (no stent) (p=0.23); UTI higher with stent 23.5% vs 5.9% (p=0.04); other outcomes NS.
ElSheemy et al. (2015) [43]	Egypt	RCT	n=90; mean 4.42 y (1–9); PCN 4.78 y; DJ 4.07 y	PCN vs Retro-DJ	PCN vs bilateral DJ	NA	Time to creatinine normalization ~2.2 d in both; overall complications 24% (PCN) vs 11% (DJ) (p=0.044); DJ reduced need for subsequent procedures vs PCN (p=0.003).
Elgammal et al. (2014) [44]	Egypt	RCT	n=66; 6.71 y vs 6.43 y	URS	Primary URS (no pre-stent) vs Secondary URS (after pre-stent)	Prestent 1–3 wk (≈10 d); postop 1–3 wk	Stone-free 95.8% (secondary URS after prestent) vs 59.5% (primary URS) (p=0.001); dilation 0% vs 73.8%; operative time ~45 vs 61 min (p<0.001).
Srivastava et al. (2017) [45]	India	RCC	n=98; mean ~8 y (3.5–14)	Transperitoneal laparoscopic ureterolithotomy	LU with stent vs stentless LU	NA	LU SFR 100% both groups; stentless had two short leaks (resolved by POD5–6); stented required anticholinergics 36% vs 4% (p<0.0001) and more analgesic (4260 vs 2440 mg, p=0.03); second GA 100% vs 0%.
Fahmy et al. (2021) [46]	Egypt	RCC	n=60; age NA	RIRS	2-week vs 4-week prestenting prior to definitive RIRS	Prestent 2 wk vs 4 wk; post-RIRS ~2 wk if placed	2- vs 4-week prestent: access 100% both, SFR 86.6% vs 90% (NS); postoperative UTI higher with 4-week (30%) vs 2-week (6.7%).
Al-Busaidy et al. (2003) [47]	Oman	RCC	n=42; mean 6.1 y (0.75–12)	ESWL	Pre-stented vs non-pre-stented	3–15 wk (mean 5.5 wk); remove 3 wk after last ESWL (GA)	SFR ~79% overall; prophylactic DJ did not change SFR but eliminated major obstructive/septic complications (0% stented vs 21% unstented, p=0.035) and shortened LOS (4.6 vs 6.4 d, p=0.022).
Rostami et al. (2012) [48]	Iran	PCC	n=50 infants; 31 d–13 mo (mean 7 mo)	ESWL	Pre-stented vs non-pre-stented	NA	Infant ESWL SFR 100% by follow-up; authors used DJ for stones >13 mm; complications low (fever 4%).
Gökçe et al. (2016) [49]	Türkiye	RCC	n=251; mean 8.9 ± 3.8 y	URS	Non-prestented pediatric URS	Prestent ≥1 wk before URS (exact dwell NA)	Overall success 80.5%; prestenting improved success 91.5% vs 77.9% (p=0.04) especially for ureteral stones and >7 mm; complications 8.5% vs 14.7% (NS); OR time ~55 vs 62 min (p=0.02).

*DJ* double-J ureteral stent, *d* days, *ESWL* extracorporeal shock wave lithotripsy, *GA* general anesthesia, *LOS* length of stay, *LU* laparoscopic ureterolithotomy, *LUTS* lower urinary tract symptoms, *min* minutes, *mo* months, *NA* not available / not reported, *OR time* operating-room time, *PCN* percutaneous nephrostomy, *PCC* prospective comparative cohort, *POD* postoperative day, *postop* postoperative, *pts* patients, *RC* retrospective cohort, *RCC* retrospective comparative cohort, *RCT* randomized controlled trial, *Retro-DJ* retrograde double-J stent, *RIRS* retrograde intrarenal surgery, *RU* renal units, *SFR* stone-free rate, *URS* ureteroscopy, *UTI* urinary tract infection, *vs* versus, *wk* weeks, *y* years

### Other endourological indications

Across other endourological indications, definitions of success varied widely and included radiological improvement, avoidance of further surgery, symptom resolution, or technical success of stent placement. Across other endourological indications, reported outcomes were heterogeneous and highly indication- and age-dependent, reflecting the exploratory and temporizing role of DJ stenting in selected pediatric populations. Evidence in this subsection was derived largely from small retrospective series and single-arm studies, with variable methodological quality and an overall moderate-to-high risk of bias.

#### Endoscopic temporization / primary hydronephrosis

Across carefully selected infants and young children with severe hydronephrosis and scintigraphic evidence of obstruction, retrograde DJ stenting was reported as a temporizing approach that enabled deferral of definitive surgery in a proportion of cases. On long-term follow-up, approximately two-thirds of renal units were reported to avoid subsequent pyeloplasty. Febrile UTI and stent dislocation were reported in a notable proportion of patients, with higher displacement rates observed in primary obstructive megaureter, and stent exchanges or removal frequently required multiple anesthetic exposures [50]. Complementing these findings, a large single-center series reported an overall resolution rate of approximately 73%, with higher rates observed in children aged ≤ 4 years. Reported complications included UTI and stent migration, and success appeared to decline with increasing age [56]. In cases where retrograde access was not feasible, percutaneous antegrade DJ stenting achieved high technical success and was associated with acceptable morbidity [55].

#### Ureterocele

Following endoscopic deroofing combined with short-term DJ stenting of approximately 4 weeks, sustained decompression and low rates of reintervention were reported in both orthotopic and ectopic ureteroceles. Post-deroofing VUR was commonly observed and was generally asymptomatic, without routine progression to ureteral reimplantation [51].

#### Primary obstructive / non-refluxing megaureter

Retrograde DJ stenting used as a first-line or temporizing approach was reported to achieve clinical resolution in approximately two-thirds of cases, with few major complications, and re-stenting was described as effective in managing some recurrences [52]. In infants, internal DJ placement across the UVJ provided urinary drainage but was associated with a high rate of reported morbidity and a substantial proportion of patients requiring subsequent surgery [53]. For short-segment primary obstructive megaureter, endoscopic balloon dilation with or without laser incision combined with DJ stenting was associated with favorable medium-term outcomes, including marked reduction in hydronephrosis and absence of reported perioperative complications [54].

#### VUR endoscopic injection

In children with solitary functioning kidneys, short-term tethered DJ placement performed approximately 2 weeks after endoscopic injection was reported to be feasible, with no acute obstruction observed, a single reported episode of febrile UTI, and favorable findings on ultrasound follow-up [57].

#### Secondary UPJO / endopyelotomy

After retrograde endopyelotomy for failed pyeloplasty, longer DJ dwell time was associated with higher reported success rates compared with shorter dwell time. In this series, a 12-week DJ was accompanied by greater reported improvement in hydronephrosis and cortical thickness than an 8-week DJ [58] (see Table 3).

**Table 3:** Detailed explanation of presented studies of other endourological indications

First author, year	Country	Study Design (RCT, cohort, case series)	Population (n), (age range)	Indication	Surgical approach	Comparison group	Stent duration (days/weeks)	Main findings
Hutflesz et al. (2023) [50]	Germany	RC	n=29 pts / 34 RU; infants; mean 5 ± 4 mo (min 1 mo)	UPJO/POM (primer HN)	Endoscopic DJ	None	Total 158 ± 80 d; per-stent 102 ± 41 d	Implant success 28/34; long-term success 68% of implanted (surgery avoided/delayed); febrile UTI 21%; dislocation 11% (higher in POM).
Chowdhary et al. (2017) [51]	India	PC	n=43 pts / 47 RU; newborn–8 y; median 11 mo	Ureterocele	Endoscopic deroofing + DJ	None	4–6 wk (plan 4 wk)	After endoscopic deroofing + DJ, secondary surgery 4.7% (orthotopic) and 15% (ectopic); no incontinence; frequent early UTIs but most resolved/downgraded.
Carroll et al. (2010) [52]	UK	RCS	n=31 pts / 38 stents; 2 mo–15 y (median 37 mo)	Megaureter	Endoscopic DJ	None	≈6 mo (some >6 mo; on antibiotics)	Technical success 95% (36/38); clinical resolution without reimplant 66%; 11/31 (35%) needed reimplant; re-stenting helped avoid surgery in 5/7.
Castagnetti et al. (2006) [53]	Italy	RCS	n=2 neonates, 8 infants, 11 renoureteral units median 3 mo	Megaureter	Endoscopic DJ ± mini-cystostomy	None	6 mo planned (2/10 early removal)	Effective temporary drainage but morbidity high (70%—UTI/occlusion/hematuria); half (50%) later needed surgery; de novo VUR in 3/10 resolved by 6 mo.
Christman et al. (2012) [54]	USA	PCS	n=17; mean 7 y (3–12); infants excluded	Megaureter	Endoscopic (balloon dilation ± laser incision) + DJ	None	8 wk	Balloon ± laser + 8-wk DJ: 71% marked hydronephrosis reduction; all asymptomatic with stable function ≥2 y; no peri-op complications.
Ključevšek and Ključevšek (2013) [55]	Slovenia	RCS	n=10; mean 9 y (1–17)	Ureteral obstruction	Percutaneous antegrade DJ	None	mean 6.4 mo (1.5–15 mo)	Antegrade DJ technical success 100% (80% first-attempt); most (7/10) avoided further surgery; complications included pyelonephritis 1, migration 1.
Pogorelić et al. (2017) [56]	Croatia	RCS	n=133; median 2 y (0–17)	Primary hydronephrosis	Endoscopic DJ	None	Median 11 months (range 10–13) before removal	Endoscopic DJ for primary HN: resolution 73% overall, best ≤4 y (83.5%), declines with age (10–14 y 33.5%, ≥15 y 0%); complications 15.8%.
Sharifi et al. (2016) [57]	Iran	RCS	n=28; mean 3.4 y (2 mo–13 y)	VUR	ECVUR + DJ	None	2 wk (one 4 wk for febrile UTI)	After ECVUR in solitary kidney, routine 2-wk DJ yielded 0% acute obstruction; 1 febrile UTI; ultrasound favorable.
Shirazi et al. (2020) [58]	Iran	RCS	n=15; median 24 mo vs 12 mo (groups)	Secondary UPJO	Endopyelotomy + DJ	8-week vs. 12-week stenting duration after endopyelotomy	8 wk vs 12 wk	After endopyelotomy for secondary UPJO, success 87.5% (12-wk) vs 57.1% (8-wk); failures 1/8 vs 3/7; one encrustation, one migration.
Koparal et al. (2025) [37]	Türkiye	RC	n=126; median 67 mo (1–201)	UPJO	Open/RALP pyeloplasty; Endo-DJ	pyeloplasty vs DJ-stent	med ~10 wk	Definitive success 43.9% (DJ-stent) vs 88.4% (pyeloplasty) (p<0.001); procedure type predicted success (OR 6.87, p<0.001); complications similar; stent preserved SRF short-term (no SRF change).

*d* days, *DJ/JJ* double-J ureteral stent, *ECVUR* endoscopic correction of vesicoureteral reflux, *Endo-DJ* endoscopic double-J stent, *HN* hydronephrosis, *min* minutes, *mo* months, *PCS* prospective case series, *POM* primary obstructive megaureter, *pts* patients, *RALP* robot-assisted laparoscopic pyeloplasty, *RC* retrospective cohort, *RCS* retrospective case series, *RU* renal units, *SRF* split renal function, *UPJO* ureteropelvic junction obstruction, *UTI* urinary tract infection, *VUR* vesicoureteral reflux, *wk* weeks, *y* years

## Discussion

In this scoping review, we mapped the contemporary evidence on the use of double-J ureteral stents across a broad spectrum of pediatric urological indications, including reconstructive procedures, stone disease, and other endourological settings. Overall, reported outcomes suggest that DJ stenting is associated with high procedural success across many indications, while clinically meaningful differences are more consistently observed in perioperative burden, anesthesia exposure, dwell time, complication profiles, and resource utilization rather than in primary efficacy outcomes. Importantly, this review highlights substantial heterogeneity in outcome definitions, study design, and methodological quality, underscoring the challenges of drawing prescriptive conclusions from the existing literature. To our knowledge, this is the first scoping review to comprehensively synthesize and contextualize these diverse data, identifying both prevailing practice patterns and critical evidence gaps in pediatric ureteral stenting.

The literature is limited by design and heterogeneity. Most data derive from retrospective, single-center cohorts with surgeon- or era-driven allocation, which can introduce confounding by indication and performance bias. Even in randomized trials, small sample sizes, lack of blinding, and short follow-up for functional or infection outcomes constrain precision and may miss late failures [16, 22, 28, 33, 42, 43].

Comparability is limited by non-standardized exposures and outcomes. “Success” variably denotes symptom relief, ultrasound improvement (APPD/SFU (*anteroposterior pelvic diameter (APPD)*; *Society for Fetal Urology (SFU) grade)*), renogram drainage, or simply no reoperation, while UTI definitions and prophylaxis differ across studies. Stent characteristics (internal vs. externalized types, 3–6 Fr calibers, length rules, ~ 1–12-week dwell, office vs. OR removal) and perioperative imaging schedules also vary, hindering pooled inference and obscuring dose–response signals (e.g., dwell-time–related infection) [13–15, 18–22, 31–32, 34, 36, 40–42, 44, 46, 49, 57–58].

In studies evaluating pediatric UNC for VUR, routine DJ stenting was reported to be associated with higher rates of short-term postoperative healthcare utilization and infectious complications compared with unstented procedures, while no consistent improvement in primary outcomes was observed [38]. In stone disease, routine pre-stenting before ESWL or RIRS does not improve stone-free rates and can raise infectious morbidity; pre-stents should be reserved for clear access/anatomic challenges (e.g., difficult ureters, huge/complex burdens), with dwell kept as short as possible (~ 2 weeks) when used [40–41–47].

Practices vary widely across centers; an international survey reported no common standard for stent placement frequency, dwell time, or stent length selection, indicating that decisions are essentially preference- and logistics-driven [59]. Pediatric data show ~ 25% DJ morbidity, driven by modifiable factors: >1 lifetime stent (odds ratio ≈ 6.6), bilateral placement (odds ratio ≈ 4.9), and 90–120 days dwell (odds ratio ≈ 6.1); routine prophylactic antibiotics did not confer protection. Protocols should minimize dwell, avoid bilateral stenting when feasible, and use robust tracking/recall systems to prevent forgotten stents and reduce complications [60].

Standardized selection was associated with reduced device-related morbidity. In children, the pragmatic “Age + 10 cm” length rule showed high predictive accuracy for correct proximal/distal curls, offering a simple starting point for sizing and a basis for local length charts [61]. Material and surface properties also influenced outcomes: propolis-coated polyurethane stents demonstrated sustained suppression of biofilm and encrustation for up to 3 months in vitro and in a rat model, suggesting a path to lower infection/encrustation burden, although pediatric clinical validation remains pending [62].

Moreover, multicenter data reported higher postoperative complication rates with externalized drainage compared to internal DJ on univariate analysis, although drainage type was not an independent predictor in multivariable models [63]. These findings support selective rather than routine use of externalized stents, with careful monitoring and standardized removal protocols.

Integrating artificial intelligence with human-factors–aware design could personalize pediatric DJ dwell and removal timing, prevent “forgotten” stents via automated tracking, and strengthen follow-up safety; high-accuracy AI frameworks proven in urolithiasis are adaptable to predict stent-related infection/encrustation risk and optimize retrieval windows. In parallel, an international consensus highlights the urgent need for pediatric-specific evidence and standardized diagnostic–therapeutic–follow-up pathways; our findings support multicenter standardization of DJ selection, dwell, and retrieval logistics in children [64–66].

Efforts to reduce anesthesia exposure must be balanced against infection control. With disciplined protocols, stent-on-string enabled office removal without increased UTIs or higher costs than OR removal, resulting in substantial cost savings [67]. Risk was concentrated in children—especially girls—with prior UTI, in whom strings were associated with higher febrile UTI [68]. Conversely, a single-center RALP series using routine strings reported more unplanned returns and pyelonephritis despite ~ 25% lower 30-day costs [12]. Net safety appears context-dependent—reliant on secure fixation, early removal, bladder management, and family education—so outcomes should be audited before scale-up.

After URS, heavy biofilm/encrustation on pediatric DJ stents appeared by 7 days—greatest on proximal/distal coils—and by 31 days, elasticity fell ~ 27–30%, increasing occlusion and device-damage risk [69]. These data support shorter dwell, early office removal when feasible, and coil-focused anti-biofilm/encrustation coatings.

Positioned as an emerging option, magnetic DJ stents can be removed in the outpatient setting without GA and have shown high retrieval success (~ 95–98%) with familiar, low complications (UTI, obstruction, distal migration). Challenges—antegrade UVJ passage in small children and 9 F retriever size—were manageable, and cystoscopic fallback maintained safety, supporting future use to reduce OR time and anesthesia exposure [70, 71].

Complications should be anticipated and managed proactively. Proximal migration is uncommon and primarily technical; prevention relies on correct stent length and a ≥ 180° distal curl. If migration occurs, retrieval is safely performed with retrograde ureteroscopy tailored to age/ureter caliber or antegrade percutaneous access [72]. Device choice also matters: 3-Fr stents in young children show ~ 20% early failure and warrant avoidance when possible or early reassessment/alternative drainage when used [73].

Perioperative resource use, sterilization, and repeat hospital visits drive procedure-level carbon footprints. Pathways that avoid an extra anesthetic and cut revisits—e.g., stent-on-string with disciplined early removal or magnetic-tip stents enabling outpatient retrieval—can lower emissions while improving turnaround time, provided infection-prevention and patient-education protocols are robust; centers should audit outcomes as they scale [12, 63–64, 74].

Several practical considerations frequently highlighted in the included studies—such as selection of smaller stent calibers, shorter dwell times, and strategies to reduce cumulative exposure to general anesthesia—should be interpreted as hypothesis-generating observations derived from the mapped literature and prevailing expert practice rather than as definitive, evidence-based recommendations. Given the predominantly retrospective nature and methodological heterogeneity of available studies, these considerations warrant prospective validation before being translated into formal clinical guidance.

This scoping review also highlights several important gaps in the current literature on pediatric ureteral stenting. Most available studies are retrospective, single-center cohorts with heterogeneous patient populations, procedural techniques, and outcome definitions, limiting comparability across indications. Standardized definitions of “success,” consistent reporting of complications, and age-stratified analyses—particularly distinguishing infants, young children, and adolescents—are notably lacking. In addition, data on long-term renal outcomes, patient-reported symptoms, quality of life, and cumulative anesthesia exposure remain sparse across most indications. Consequently, the interpretability and strength of the mapped associations are constrained by the predominance of retrospective designs and moderate-to-high risk of bias and should therefore be viewed as reflecting the best available evidence rather than high-level comparative data. Age-related heterogeneity represents an additional limitation, as several studies combined infants, school-aged children, and adolescents without stratified analysis, limiting age-specific inference.

Future research should prioritize prospective, multicenter comparative studies using standardized outcome measures to better delineate the role of DJ stenting across specific pediatric urological indications. Particular attention should be directed toward defining optimal stent characteristics, dwell duration, and removal strategies, and to identifying subgroups most likely to benefit from stenting versus stentless approaches. Incorporation of patient-centered outcomes, cost-effectiveness analyses, and long-term follow-up will be essential to inform evidence-based guidance and reduce unwarranted variation in clinical practice.

Across reconstructive indications, internal DJ and externalized stents demonstrate broadly comparable reported success rates, with differences primarily relating to removal logistics and cumulative anesthesia exposure rather than efficacy. In ureteroneocystostomy and stone disease, routine stenting or pre-stenting is not consistently supported by the currently available literature. Shorter dwell times appear to be associated with fewer stent-related complications across indications, and strategies aimed at reducing repeated exposure to general anesthesia may offer practical advantages, although high-level prospective validation remains limited. Overall, substantial heterogeneity in outcome definitions, study design, and reporting standards continues to limit direct comparability across pediatric studies.

## Conclusion

This scoping review demonstrates that reported outcomes of pediatric DJ stenting vary substantially by indication, technique, dwell time, and removal strategy, with consistently high procedural success across many settings but heterogeneous complication profiles and perioperative burden. For reconstructive surgery, internal double-J and externalized stents provide comparable success; externalized or tethered options can reduce anesthesia exposure yet may carry higher minor complication risks in some series, underscoring the importance of individualized selection, protocolized dwell time, and local outcome audit. In stone disease, the mapped literature suggests that routine pre-stenting is not consistently associated with improved stone-free outcomes, while several studies report higher infectious morbidity, highlighting an area of ongoing clinical uncertainty. Future research should focus on standardizing stent selection, dwell time, and reporting, while evaluating innovations such as magnetic or coated stents. Collaborative multicenter data are crucial to define safe, cost-effective, and child-centered stent strategies. These conclusions reflect patterns identified in the currently available literature and should be interpreted within the context of predominantly non-randomized and heterogeneous evidence.

## Supplementary Information

Below is the link to the electronic supplementary material.

Supplementary Material 1

## Data Availability

No datasets were generated or analysed during the current study.
