# Supplementary material for: Pediatric ureteral stenting: state-of-the-art review
Source: World J Urol. 2026 Apr 9;44(1):289. doi: 10.1007/s00345-026-06347-8 (PMC13065543; doi:10.1007/s00345-026-06347-8)
Supplement: Supplementary file 1 — Supplementary Material 1 [file 345_2026_6347_MOESM1_ESM.docx]

Complete Search Strategies

Search Date

All databases were searched from inception to 9 September 2025, without date restrictions. Searches were limited to human studies. No language restrictions were applied at the search stage; language eligibility was assessed during screening.

1- PubMed (MEDLINE)

Search performed: 9 September 2025
(("Ureteral Stents"[Mesh] OR "ureteral stent"[tiab]

OR "ureteric stent"[tiab] OR "double J stent"[tiab] OR "DJ stent"[tiab])

AND

("Child"[Mesh] OR "Infant"[Mesh] OR "Adolescent"[Mesh]

OR child*[tiab] OR children[tiab] OR pediatric*[tiab] OR paediatric*[tiab] OR adolescent*[tiab] OR infant*[tiab]))

Number of records retrieved: 868

2- Scopus

Search performed: 9 September 2025
(TITLE-ABS-KEY("ureteral stent"

OR "ureteric stent" OR "double J stent" OR "DJ stent"))

AND

(TITLE-ABS-KEY(child*

OR pediatric* OR paediatric* OR adolescent* OR infant*))
Number of records retrieved: 1,252

3- Cochrane Library

Search performed: 9 September 2025
("ureteral stent" OR "ureteric stent" OR "double J stent" OR "DJ stent")

AND

(child* OR pediatric* OR paediatric* OR adolescent* OR infant*)
Number of records retrieved: 111

4- Web of Science Core Collection

Search performed: 9 September 2025
TS=("ureteral stent" OR "ureteric stent" OR "double J stent" OR "DJ stent")

AND

TS=(child* OR pediatric* OR paediatric* OR adolescent* OR infant*)
Number of records retrieved: 780

5-Manual Search

An additional five studies were identified through manual reference screening of included articles and relevant review papers.
